# Interpreting the decisions of CNNs via influence functions

**DOI:** 10.3389/fncom.2023.1172883

**Published:** 2023-07-26

**Authors:** Aisha Aamir, Minija Tamosiunaite, Florentin Wörgötter

**Affiliations:** ^1^Third Institute of Physics – Biophysics and Bernstein Center for Computational Neuroscience, University of Göttingen, Göttingen, Germany; ^2^Department of Informatics, Vytautas Magnus University, Kaunas, Lithuania

**Keywords:** deep neural networks, interpretability, layer-wise, influence score, explainable AI

## Abstract

An understanding of deep neural network decisions is based on the interpretability of model, which provides explanations that are understandable to human beings and helps avoid biases in model predictions. This study investigates and interprets the model output based on images from the training dataset, i.e., to debug the results of a network model in relation to the training dataset. Our objective was to understand the behavior (specifically, class prediction) of deep learning models through the analysis of perturbations of the loss functions. We calculated influence scores for the VGG16 network at different hidden layers across three types of disturbances in the original images of the ImageNet dataset: texture, style, and background elimination. The global and layer-wise influence scores allowed the identification of the most influential training images for the given testing set. We illustrated our findings using influence scores by highlighting the types of disturbances that bias predictions of the network. According to our results, layer-wise influence analysis pairs well with local interpretability methods such as Shapley values to demonstrate significant differences between disturbed image subgroups. Particularly in an image classification task, our layer-wise interpretability approach plays a pivotal role to identify the classification bias in pre-trained convolutional neural networks, thus, providing useful insights to retrain specific hidden layers.

## 1. Introduction

Machine learning algorithms based on deep networks have outperformed humans in solving tasks in various fields, not only in the computer vision domain but also in the industrial and medical fields (Chmiela et al., 2018). Furthermore, they exhibit exceptional abilities when it comes to making predictions, analyzing data, and presenting visualizations (Thomas et al., 2019; Wu et al., 2019). The success of deep networks is due to the availability of high-end computing devices (Lindholm et al., 2008), large datasets for learning (Deng et al., 2009; Karpathy et al., 2014), and improved deep learning techniques (LeCun et al., 2012, 2015). Despite their success in many domains, these complex structures suffer from a lack of interpretability and transparency of their learned representations. The main reason for this may be attributed to their “black-box” nature and the distributed encoding of the data on which they generalize and learn representations (Samek et al., 2017). For understanding the input-output relationship of these complex models (Fernandez et al., 2019), it is necessary to probe the individual or cluster of neurons to visualize and encode the acquired concepts (Li et al., 2016; Bau et al., 2017). It is possible to construct prototypes of learned representations in an abstract manner using certain approaches. For example, these methods learn representations of classes of interest by creating prototype images (Simonyan et al., 2014; Yosinski et al., 2015; Nguyen et al., 2016, 2019). In general, these types of learned representations are based on activation maximization and have proved to be effective tools for providing a more transparent and visual understanding of CNNs. It is also possible to make CNN decision-making transparent by considering individual predictions, i.e., by highlighting the most relevant pixels on a heatmap (Simonyan et al., 2014; Montavon et al., 2018). The authors of Bach et al. (2015) and Montavon et al. (2019) used layer-wise relevance propagation to explain predictions applicable to CNNs, LSTMs (Arras et al., 2017), and support vector machines (Kauffmann et al., 2018). A spectral relevance analysis is presented by Lapuschkin et al. (2019) to understand predictions based on model behavior. This analysis identifies individual heatmaps and clusters the learned concepts for classification.

There is a long history of influence functions in statistics, but in the literature, there is little information regarding their application in deep learning. In statistics, influence functions measure the change in a parametric observation and its effect on an estimator, making them useful for comparing the robustness and bias of an estimator (Fisher and Kennedy, 2018). It is possible to use this notion in the domain of deep learning to debug the results of a network model in relation to the training dataset. This can be simplified by determining if the network has a loss function that is twice differentiable with respect to its parameters. In such a case, we can approximate the influence of any instance on the model parameters. Additionally, influence functions have not been widely used in machine learning models due to the high computational cost of determining whether a model’s loss function is twice differentiable. In spite of this, there are methods for approximating influence functions in an efficient and accurate manner using second-order optimization techniques (Martens, 2010; Agarwal et al., 2017). Furthermore, Koh and Liang (2017) and Khanna et al. (2018) use influence functions to provide example-based explanations by identifying the most influential training images. As a result, these methods are useful for identifying model errors and biases, identifying mislabeled datasets, and debugging models, but they lack interpretability when it comes to identifying the learned representations. A second-order optimization technique is also used by Koh and Liang (2017) to approximate Influence functions in order to represent the behavior of the model based on training data.

Since, CNNs are black boxes there is no transparent way to identify how these complex models make classification decisions. Therefore, in this paper we tried to explore and interpret the individual layers and provide this way an extension to the work by Koh and Liang (2017) which had previously only explored global parameters to identify influential training images. The work we present goes beyond the previous work in sense that we now give a better in depth understanding of the relevance of the individual layers of this network. Layer-wise analysis is more efficient in identifying biases in the network decisions for which we gave examples through our texture, styled analysis and background elimination experiments. Our approach helps making the behavior of individual layers more transparent and identifies which layers can be retrained to overcome biases in decisions. Interpreting individual layers of the network and making transparent representations via influential images has not been performed previously. In this study, we investigate and interpret model output based on images in the training dataset, i.e., what characteristics of the training images influence the class predictability of the network. To do this, we calculate layer-wise influence scores for each training image in relation to each test image, to determine which features of the training image are most influential at each layer. We add three types of disturbance to the input images: texture, style, and background removal in order to determine to what degree these disturbances contribute to class prediction. The following are our main contributions in this paper:

• An approach based on layer-wise analysis is proposed to determine influence scores and influential training images that contribute to class prediction.
• Through our layer-wise interpretability, we can gain a deeper understanding of the black box model’s hidden layers.
• To interpret network predictions, we provide a bi-directional interpretability approach, which includes training images (using influence scores) and testing images (using Shapley values).

Layer-wise influence analysis of the disturbed images subgroups can be an effective method of studying and providing transparent solutions to the abstract representations of a network model.

## 2. Related work

There are several challenges associated with the choice of an appropriate method which yields insights into deep network performance, but one needs to be sure that the description of these methods reflects the internal functionality of the models (Goodman and Flaxman, 2017). A more transparent explanation is needed for the predictions of even the highest performing deep learning models in various computer vision domains. The impact of perturbing data points on interpretability has been extensively examined by Adler et al. (2016), Datta et al. (2016), Li et al. (2016), Koh and Liang (2017), Lundberg and Lee (2017) and Kindermans et al. (2018) and they evaluated the effect on model outcomes either globally or locally. Furthermore, perturbation-based methods are often inconsistent in their explanations, which could be true for one data point but not for its neighboring points, or for similar data points within the same class.

The saliency-based method is mostly used to interpret local features in image classification tasks (Erhan et al., 2009; Selvaraju et al., 2016; Dabkowski and Gal, 2017). These methods emphasize the importance of individual pixels in image classification tasks; however, conclusions drawn from one image cannot be applied to another image. Hence, these local explanations do not adequately reflect model decisions. We should instead develop methods that address the distributed encoding of a neural network in a systematic manner. In this regard, influence functions are a technique that originated in statistics and has been used in machine learning tasks in order to track predictions back to training data (Koh and Liang, 2017) and to investigate robustness and cross-validation within a model (Christmann and Steinwart, 2004; Debruyne et al., 2008; Liu et al., 2014). The Cook’s distance is estimated using a similar method for prioritizing the training points (Wojnowicz et al., 2016), whereas an influence-based distance metric is used to configure classifiers (Kabra et al., 2015). There are also other methods that utilize influence functions in which adversarial examples are used to interpret the decisions of the model (Goodfellow et al., 2014). Another work in this regard, performed by Moosavi-Dezfooli et al. (2016), also deals with training examples with adversarial attacks. Furthermore, changing the labels of the classes in the subset of the training set improves the performance of the network for incorrect test inputs (Cadamuro et al., 2016). Although much work has been done using adversarial perturbation as given in the work of Gu and Rigazio (2014), Szegedy et al. (2014), and Nguyen et al. (2015) to trick the convolutional neural networks in various classification tasks, still there are many aspects unexplored. In different machine learning models, these methods performed well and they are now being used in deep learning as robust statistical approaches for *post hoc* interpretation. Here, we are using influence functions to determine which apparent disturbances in images are most sensitive to the network, and we examine how the hidden layers differ in terms of their influence scores in the presence of different types of disturbances.

## 3. Materials and methods

### 3.1. Influence score and data set

Let us assume that the CNN is pre-trained for the task of image classification, and we want to find out the importance of different hidden layers. The non-linear nature of CNNs gradually untangles the semantic information as the activation moves toward the deeper layers (Alain and Bengio, 2016; Bau et al., 2017; Raghu et al., 2017). Specifically in this scenario, we will use Influence functions (Koh and Liang, 2017) to analyze network decisions in case of regular test set images as well as when disturbances have been added to those images.

To accomplish this, we first define a training dataset for a neural network as *R*_*t*_ = {*x*_1_, *x*_2_, …, *x*_*n*_}, where *n* is the number of training samples; *x*_*i*_ = (*a*_*i*_, *b*_*i*_) where *a_i_* are class images of the size 224 × 224 provided to the input of the neural network, and *b*_*i*_ is the true class label of the network, defined as one-hot encoding in *c* output lines, where *c* is the number of classes. We then define a loss function given as:


(1) $$L\,\left(\theta \right)=\frac{1}{n}\,\sum_{i=1}^{n}L\,\left(x_{i},\theta \right)$$

Where *L* is the categorical-cross entropy loss with which the network was pre-trained, i.e., a softmax followed by cross entropy loss written as: $L=-\sum_{i=1}^{c}b_{i}\,l\,o\,g\,\left(f\,{\left(a\right)}_{i}\right)$ where *f*(*a*)_*i*_ is the probability of each class and *b*_*i*_ encodes the true class through one-hot encoding.

Let us say that all learning samples in the beginning are contributing to the loss equally with coefficients 1n as defined in eq. (1) above. We will probe the loss function *L*(θ) by decreasing contributions of individual learning samples. Specifically, we will investigate the perturbation of the loss with respect to the training samples. Our aim is to calculate how the network parameters θ would change in the case of changing the contribution of a specific sample *x_j_* ∈ (*j* = 1, 2, …*n*) to a loss by a small quantity *ϵ*. For that, first we need to evaluate optimal network parameters for the loss function with the perturbation:


(2) $${\hat{\theta }}_{\epsilon ,x_{j}}\overset{\text{def}}{=}\arg m\,i\,n_{\theta \in \Theta }\,\frac{1}{n}\,\sum_{i=1}^{n}L\,\left(x_{i},\theta \right)+\epsilon \,L\,\left(x_{j},\theta \right)$$

In the work of Koh and Liang (2017), it was shown that the rate of change in optimum network weights $\hat{\theta }$ with respect to *ϵ* the way the latter quantity is defined in eq. (2), under the assumption of quadratic approximation of the loss function, can be expressed as follows:


$$I_{mod,params}(x_{j})=\frac{d{\hat{\theta }}_{\epsilon ,x_{j}}}{d\epsilon }{|}_{\epsilon =0}$$

(3) $$=-H_{\hat{\theta }}^{-1}\,\nabla_{\theta }L\,\left(x_{j},\hat{\theta }\right)$$

Where, *I*_*mod*,*params*_ (*x*_*j*_) is image with modified parameters, $\nabla_{\theta }L\,\left(x_{j},\hat{\theta }\right)$ is the perturbation of approximated loss gradient with respect to the training sample at the point $\left(x_{j},\hat{\theta }\right)$ and $H_{\hat{\theta }}$ is defined as follows:


(4) $$H_{\hat{\theta }}=\frac{1}{n}\,\sum_{i=0}^{n}\nabla_{\theta }^{2}L\,\left(x_{i},\hat{\theta }\right)$$

Let us now define the test set (including both original test images and images with disturbances) as $R_{c}=\left\{{\hat{x}}_{1},{\hat{x}}_{2},...,{\hat{x}}_{m}\right\}$. We will calculate the influence of the training image *x*_*j*_ on the loss at the test image ${\hat{x}}_{k}$ following the approximation given in Koh and Liang (2017):


$$I_{m\,o\,d,l\,o\,s\,s}\,\left(x_{j},{\hat{x}}_{k}\right)={\frac{d\,L\,\left({\hat{x}}_{k},{\hat{\theta }}_{\epsilon ,x_{j}}\right)}{d\,\epsilon }|}_{\epsilon =0}$$

$$={\nabla_{\theta }L\,{\left({\hat{x}}_{k},\hat{\theta }\right)}^{T}\,\frac{d\,{\hat{\theta }}_{\epsilon ,}\,x_{j}}{d\,\epsilon }|}_{\epsilon =0}$$

(5) $$=-\nabla_{\theta }L\,{\left({\hat{x}}_{k},\hat{\theta }\right)}^{T}\,H_{\theta }^{-1}\,\nabla_{\theta }L\,\left(x_{j},\hat{\theta }\right)$$

Since directly computing the Hessian matrix $H_{\hat{\theta }}$ and its inverse as given in Eqs. (3–5) is computationally expensive, we used stochastic gradient to obtain Hessian Vector Products (HVP’s) and their inverse on mini batch of training images. Specifically, we calculate HVP’s for each layer, which is the product between the Hessian matrix $H_{\hat{\theta }}$ and gradient vector of loss.

In the work of Koh and Liang (2017) the gradients in Eq. (3) and influential image in Eq. (5) were calculated based on the entire parameter set θ of the neural network. We expand this approach, by performing the analysis layer-wise, by separately finding gradients and HVPs for each layer *l* in the network, thus obtaining layer-wise influence scores as given below:


$$I_{s\,c\,r\,\_\,l}\,\left(x_{j},{\hat{x}}_{k}\right)=I_{m\,o\,d,l\,o\,s\,s}^{l}\,\left(x_{j},{\hat{x}}_{k}\right)={\frac{d\,L\,\left({\hat{x}}_{k},{\hat{\theta }}_{\epsilon ,x_{j}}^{l}\right)}{d\,\epsilon }|}_{\epsilon =0}$$

(6) $$=\left(-\nabla_{\theta }L\,{\left({\hat{x}}_{k},\hat{\theta }\right)}^{T}\,H_{\hat{\theta }}^{-1}\,\nabla_{\theta_{i}^{l}}L\,\left(x_{j},{\hat{\theta }}_{\epsilon ,x_{j}}^{l}\right)\right)$$

In the above expression, ${\hat{\theta }}^{l}$ represents the parameters for the *l^th^* layer of the network, $\theta_{i}^{l}$ is the *i^th^* parameter for a specific layer for which the inverse hessian is being calculated and *I*_*scr_ l*_ is the influence score for a specific layer. The computation for Eq. (6) can be efficiently obtained via TensorFlow expressions given later in the text that will return the layer-wise gradients of the loss $L\,\left({\hat{\theta }}_{i}\right)$ for an image *x*_*j*_, which we denote as $\nabla_{\theta_{i}^{l}}L\,\left(x_{j},{\hat{\theta }}_{\epsilon ,x_{j}}^{l}\right)$. By varying the parameter indices of $\nabla_{\theta_{i}^{l}}L\,\left(x_{j},{\hat{\theta }}_{\epsilon ,x_{j}}^{l}\right)$ in the above equation we can extract the influence scores for each individual layer. To calculate the layer-wise score, we will be evaluating expression (6) for each possible triplet of a training set image, network layer, and test set image $\left(x_{j},{\hat{x}}_{k}\right)$, j = 1, 2, …n and k = 1, 2, …m and will call it influence score. The image in the training set *x*_*j*_ with the highest influence score for the test set image ${\hat{x}}_{k}$ will be called the (most) influential image in layer *l* and can be formally defined as: $I_{\inf}\,\left(l,{\hat{x}}_{k}\right)=\arg m\,a\,x_{j=1,2..n}\,I_{m\,o\,d,l\,o\,s\,s}^{l}\,\left(x_{j},{\hat{x}}_{k}\right)$. An overall layer-independent (most) influential image can also be obtained using the expression in Eq. (5) as $I_{\inf}\,\left({\hat{x}}_{k}\right)$$=\arg m\,a\,x_{j=1,2..n}\,I_{m\,o\,d,l\,o\,s\,s}\,\left(x_{j},{\hat{x}}_{k}\right).$

We also analyze the compound influence based on layer-wise influence scores:


(7) $${\text{I}}_{t\,o\,t\,a\,l}\,\left(x_{j},{\hat{x}}_{k}\right)=\sum_{l=1}^{m}I_{s\,c\,r\,\_\,l}\,\left(x_{j},{\hat{x}}_{k}\right)$$

It is known that computing a second order derivative of a hessian matrix and its inverse, especially when the matrix constitutes of optimizing the parameters as in case of deep neural network, becomes very expensive in terms of space and time.

We use the TensorFlow implementation of *get*_*Hv*_*op*() defined in *pyhessian* as: (*Hv* = flatten (*tf*.*gradients*(*tf*.*math*.$\text{multiply}\left(\mathrm{flatten}\left(\mathrm{tf}.\mathrm{gradients}\left(\text{L},{\hat{\theta }}_{i}\right)\right),\text{tf}.\text{stopgradient}\left(v\right)\right),$*params*))) to efficiently calculate the HVP’s similar to the one used by Koh and Liang (2017). This efficiently reduced the computational cost and made this approach feasible. We save the influence score of each layer into associated arrays or dictionaries and extract the influential image based on the highest influence score for the purpose of analysis. In addition, we take the inter and intra-class mean of layer-wise influence score for each test set image with disturbance and save the score in the dictionaries. Whereas, the total influence for a particular image ${\hat{x}}_{k}$ is the sum of all layer-wise influences calculated in Eq. (7) as the compound influence score. Later, we show a comparison and establish a relationship of the intra-class mean influence score calculated for a particular test image with disturbances, using I_*total*_ from Eq. (7), and the corresponding non-disturbed image as a control group. In this study, we have taken an average of the intra-class and inter-class influence score over the samples within each class given as: $\,I_{a\,v\,g}=\frac{\,\sum I_{t\,o\,t\,a\,l}\,\left(f\,o\,r\,\,e\,a\,c\,h\,\,i\,m\,a\,g\,e\,\,p\,e\,r\,\,c\,l\,a\,s\,s\right)}{N\,o\,\,o\,f\,\,i\,m\,a\,g\,e\,s\,\,p\,e\,r\,\,c\,l\,a\,s\,s}$ to identify an influence based trend between the image disturbance and their controlled groups.

**FIGURE 1:**
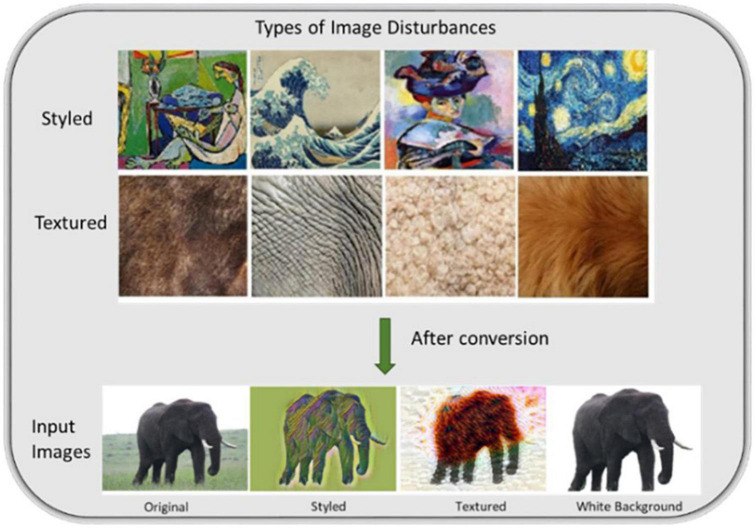
Exemplary styled and textured images used in the dataset for adding disturbances. Styled images are taken from the work of famous artists (left to right: The Muse by Pablo Picasso, 1935; The Great Wave off Kanagawa, 1831; Woman with a Hat (Femme au chapeau) by Henri Matisse, 1905; The Starry Night by Vincent van Gogh, 1889). As an example of the dataset used in the analysis, an original image of elephant class is shown with added disturbances.

In order to test our methodology, we used the VGG16 network architecture, and defined the cues we wanted to quantify. We evaluated our method on the ImageNet dataset with 10 classes (chair, cat, elephant, zebra, screwdriver, bird, cup, toaster, bus and bicycle) using three types of disturbances: (1) foreground placed on a white-background, (2) texture added (Textured) and (3) style added (Styled), where original images were used as controls (Figure 1). We simplified the class labeling and do not categorize with a separate class label, i.e., ImageNet’s Tabby cat/Persian cat is labeled as “cat” and humming bird/Goldfinch as “bird,” etc. All original images are from the ImageNet dataset, and we used a subset of approximately 30,000 images (30 × 1000) as our training set and 200 images as a testing dataset, where the latter contains disturbances (200 = 5 images × 10 classes × (1 original + 3 disturbance types)).

For white-background images (five per class) we eliminated the background and replaced it with white color, whereas, for styled and textured images we adopted the method from Gatys et al. (2015) and Johnson et al. (2016), respectively. We transferred different styles and texture to 50 images (5 per class) added to the white-background images.

### 3.2. Clustering and cluster variables

We used hierarchical clustering as a statistical method to determine the similarity among the various images based on the observed disturbances. We started by considering the images with added disturbances and the inter/intra-class control images of the VGG16 as separate clusters, i.e., a cluster of singletons. We took an average of the individual layers from (*I*_*l*_*avg*_ = *l*_1_*avg*_, …, *l*_16_*avg*_) for all the class images in the testing dataset (i.e., 5 images × 4 cases × 10 classes) as feature vectors. The clustering was based on 4 cases: original image, image with white background, textured and styled images. At each iteration step, we selected two singletons (i.e., images with disturbances and original images) and measured the similarity *S*_*ij*_ (%) between cluster-singletons "*i*" and "*j*.” We used average linkage to calculate Euclidean distance *d*_*ij*_ between singletons from cluster-variables (i.e., original, styled, textured and white background) and merged the pair with the least distance. We then calculated a correlation distance matrix using Pearson’s correlation ρ_*ij*_ (Marti et al., 2017) to identify the common characteristics based on the image feature vectors.

## 4. Results

Our experiments were conducted on the VGG16 architecture using the ImageNet dataset, with our training set consisting of 30,000 images. To show results for the network model’s transparency in decisions, we calculated layer-wise influence scores for 200 test set images. Some exemplary images used in the analysis can be found in the Supplementary material.

### 4.1. Image classification with input image disturbances

In an initial experiment, we examined the classification response to all types of disturbances. Almost all images from the original and white-background categories were correctly classified by the network (Table 1). The accuracy values of the images shown in Table 1 are the Top 5 probability values of the pre-trained VGG 16 network. It is noteworthy that images with a white background showed comparable predictions to images in the original category, making the object an important cue for decision making. There was a slight decrease in prediction probabilities for the styled images when compared to the original and white-background images. By contrast, textured images did not get correctly classified because the texture was derived from a completely different class of images. Therefore, the decision was biased because it was based on the added texture rather than the class itself, suggesting that the texture of the image plays an important role in network decision-making.

**TABLE 1:** Classification accuracies and standard error of means (SEM) showing the results for five classes of ImageNet.

Type of images	Class label	Accuracy	SEM
Original	Bicycle	0.50	± 0.23
	Birds	0.95	± 0.04
	Bus	0.97	± 0.01
	Cat	0.54	± 0.17
	Elephant	0.71	± 0.03
Styled	Bicycle	0.20	± 0.02
	Birds	0.29	± 0.09
	Bus	0.79	± 0.10
	Cat	–	–
	Elephant	–	–
Textured	Bicycle	–	–
	Birds	–	–
	Bus	–	–
	Cat	–	–
	Elephant	–	–
White background	Bicycle	0.34	± 0.05
	Birds	0.92	± 0.07
	Bus	0.97	± 0.01
	Cat	0.50	± 0.17
	Elephant	0.71	± 0.10

All original images were pre-trained on VGG16 and were classified in a range between (50 and 97%) with correct class labels. As for the styled images classification accuracies mostly fluctuate between (20 and 79%), images with white background (34–97%). However, all textured images failed to give correct class predictions (0%) hence are not listed in the table above.

### 4.2. Influential image scores and class predictions

In the previous sections, we showed how to calculate influence scores for each of the 16 layers of the VGG16 network and named it layer-wise influence score (*I*_*scr_ l*_). We also defined the total influence score *I*_*total*_ as the sum of all the 16 layers to analyze the global network predictions. To identify influential images, here we used this total influence score for all images with added disturbances as well as the original images to identify which training image influenced the prediction of a testing image with the highest influence score.

**FIGURE 2:**
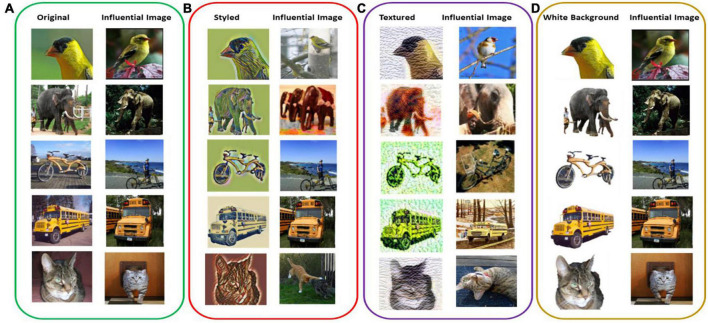
Intra-class influential images with highest influential score calculated using (*I*_*total*_) among the training set and corresponding test inputs of panels **(A)** original, **(B)** styled, **(C)** textured, and **(D)** white background images.

**FIGURE 3:**
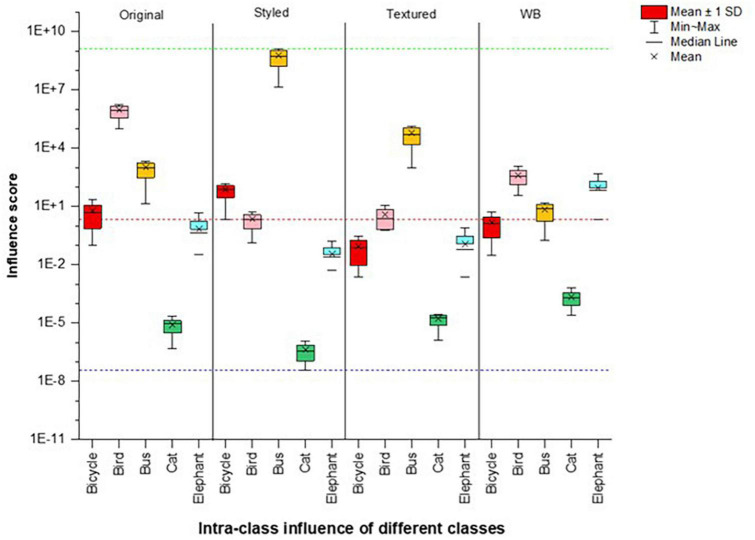
Comparison of intra-class scores showing a plot between average influence scores (*I*_*avg*_) of original, styled, textured images and white background images for five classes. Red dotted reference line indicates median whereas green and blue lines indicate max and min ranges for the spread of the whole dataset, respectively.

All the original images were predicted correctly and were given the same predicted class label as their influential image (Figure 2A and Table 2). For the white-background images, the network’s prediction was also correct, and the influential training image also belonged to the same class label as the original (Figure 2D). As for the styled and textured images, one interesting observation is the similarity of the spatial features of the test images with their most influential training images (Figures 2B, C). However, in this case, the influential images played a negative role in the sense that the predicted class labels for the test images were very different from their influential image class, indicating a strong dependence on the styling and texture representations of the input images (Table 2). Also, for styled and white-background images, the average influence score shows comparable fluctuations with their original images as control groups, indicating their strong influence on correct network classifications, as is also evident from Table 1. By contrast, we observe a larger dispersion of influence score in images that have been styled or textured (Figure 3), because their visual appearance has been disturbed. However, the average influence score of all textured images as well as their control group in most cases are very different, indicating that influential training images are not considered for prediction, but the added texture was more dominant in network decisions (Table 2).

**TABLE 2:** Intra-class influential images and the corresponding test images with their ImageNet class id and class label along with average influence score shown for five classes.

Types of images	Influential image		Test image		Average influence score
	Class id	Class label	Class id	Class label	
Original	11	“Goldfinch, Carduelis”	11	“Goldfinch, Carduelis”	4.73E + 05
	385	“Indian elephant, Elephas maximus”	385	“Indian elephant, Elephas maximus”	4.58E + 00
	444	“Bicycle-built-for-two, tandem bicycle”	444	“Bicycle-built-for-two, tandem bicycle”	2.22E + 01
	779	“School bus”	779	“School bus”	1.83E + 03
	281	“Tabby, tabby cat”	281	“Tabby, tabby cat”	1.13E−03
Styled	11	“Goldfinch, Carduelis”	88	“Macaw”	3.83E + 00
	385	“Indian elephant, Elephas maximus”	917	“Comic book”	1.32E + 01
	444	“Bicycle-built-for-two, tandem bicycle”	671	“Mountain bike, all-terrain bike”	1.07E + 02
	779	“School bus”	779	“School bus”	1.03E + 09
	281	“Tabby, tabby cat”	557	“Flagpole, flagstaff”	3.45E−05
Textured	11	“Goldfinch, Carduelis”	386	“African elephant, Loxodonta africana”	1.18E + 01
	385	“Indian elephant, Elephas maximus”	184	“Irish terrier”	7.90E−01
	444	“Bicycle-built-for-two, tandem bicycle”	533	“Dishrag, dishcloth”	2.47E−01
	779	“School bus”	533	“Dishrag, dishcloth”	1.08E + 05
	281	“Tabby, tabby cat”	386	“African elephant, Loxodonta africana”	2.87E−04
White background	11	“Goldfinch, Carduelis”	11	“Goldfinch, Carduelis”	3.84E + 01
	385	“Indian elephant, Elephas maximus”	385	“Indian elephant, Elephas maximus”	4.91E + 02
	444	“Bicycle-built-for-two, tandem bicycle”	444	“Bicycle-built-for-two, tandem bicycle”	4.95E + 00
	779	“School bus”	779	“School bus”	1.25E + 01
	281	“Tabby, tabby cat”	281	“Tabby, tabby cat”	8.32E−04

The varying influential image class id and label for styled and texture images indicate a strong dependence on the added visual disturbance.

**FIGURE 4:**
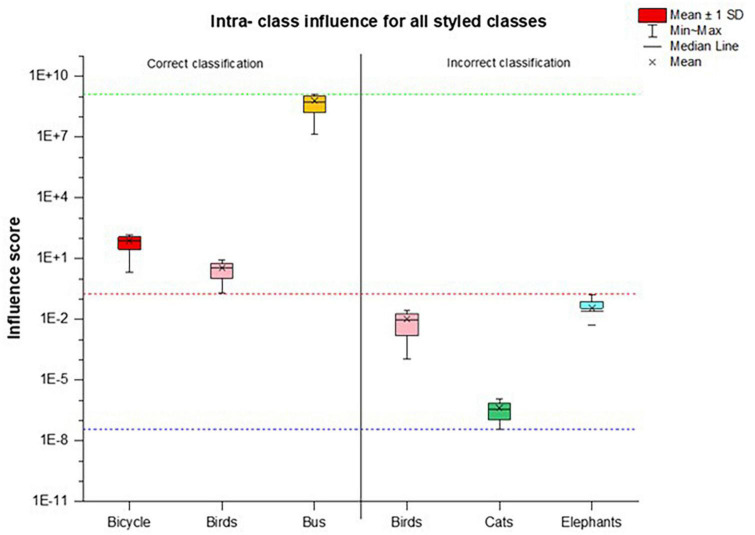
A dataset split of five intra-class styled images with correct and incorrect classification results. The images with influence score in a positive median range are classified correct while all images with influence score range below the median are incorrectly classified. Red dotted lines show the median, whereas the green and blue line show the min-max range of influence scores of the whole dataset.

We calculated the average of the intra-class influence score (*I*_*avg*_) to show a comparison of the images with disturbances with their original images as control plotted for five classes in Figure 3. The images were correctly classified if the influence score range lies at or above the positive median range of the intra-classes. Since most styled classes were classified correctly, we split the dataset to give a clearer distinction of the network behavior as shown in Figure 4 for the aforementioned classes. We can see that all images of the Bicycle and Bus class were correctly predicted by the network, whereas for the Birds class only the images with influence score above the median range of dataset were correctly classified. All those images that do not lie in this range and their influence score is low were misclassified.

#### 4.2.1. Analysis on images with texture

**FIGURE 5:**
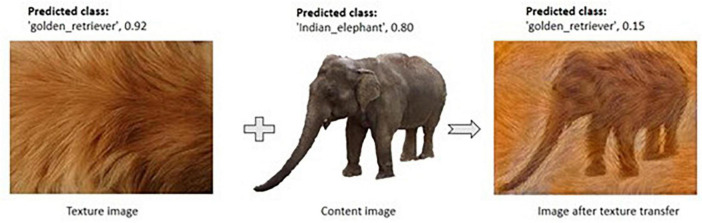
Example showing predicted class label and accuracy for elephant class before and after transfer of texture.

To verify the importance of texture for the network’s decisions, we tested the network with texture patches as input. The network was able to correctly identify the texture with classification probabilities given in Table 3. Further, we transferred the textured patches to 50 white-background images using the method Gatys et al. (2015) (Figure 5) to confirm our findings. Again, the network was making its decision based on the texture but not on the content of the image (Table 3, second column).

**TABLE 3:** Classification accuracies of texture image patches and class labels of content images after texture transfer.

Before texture transfer		After texture transfer	
**Texture image patch**	**Accuracy**	**Content image**	**Predicted class label**
Zebra	0.93	Bird	Zebra
Leopard	0.90	Cat	Leopard
Elephant	0.95	Bus	Indian elephant
Golden retriever	0.92	Elephant	Golden retriever

**FIGURE 6:**
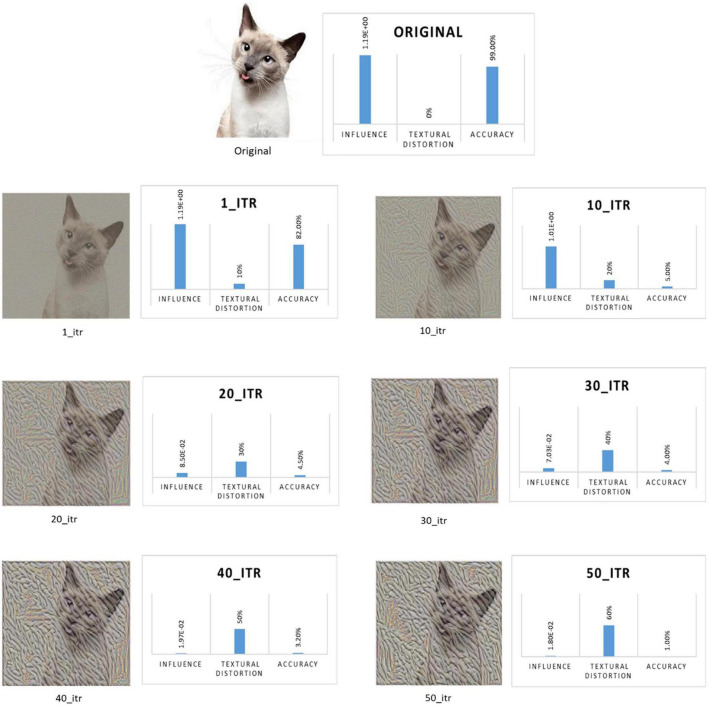
An example showing elephant texture patch transfer for 50 iterations on an image of a cat class showing the decrease of influence score and accuracy with the increase of texture at different iteration steps.

As a case study, we further evaluated the textural biasness by adding texture with different percentages onto the images, hence, checking the correct prediction capability of the network model. We show the results of this case study example by using elephant texture patches applied with various percentages to an image of the Cat class (Figure 6). Maximum image distortion starts as soon as the texture transfer algorithm reaches the 10th iteration and continues up to iteration 20. Then it gradually increases with the remaining transfer steps. During this time the appearance of the original images changes in a drastic manner making it difficult for the network to correctly identify the predicted class label. The change in the training image class label was observed to vary after the 10th iteration step of the texture transfer. The influence score shown here in the example is calculated with respect to intra-class of the cat class.

A change in the amount of added texture (%) affected the network’s decisions in an inverse proportion to the fluctuation in influence scores. In addition, the amount of texture on the test images decreased the influence that a training instance had on the network’s ability to correctly classify the images. Consequently, incorrect object recognition resulted as the texture on test images increased and the influence score became negative. Thus, the image’s texture has a greater influence on the network’s decisions than the object itself.

#### 4.2.2. Analysis on images with style

**FIGURE 7:**
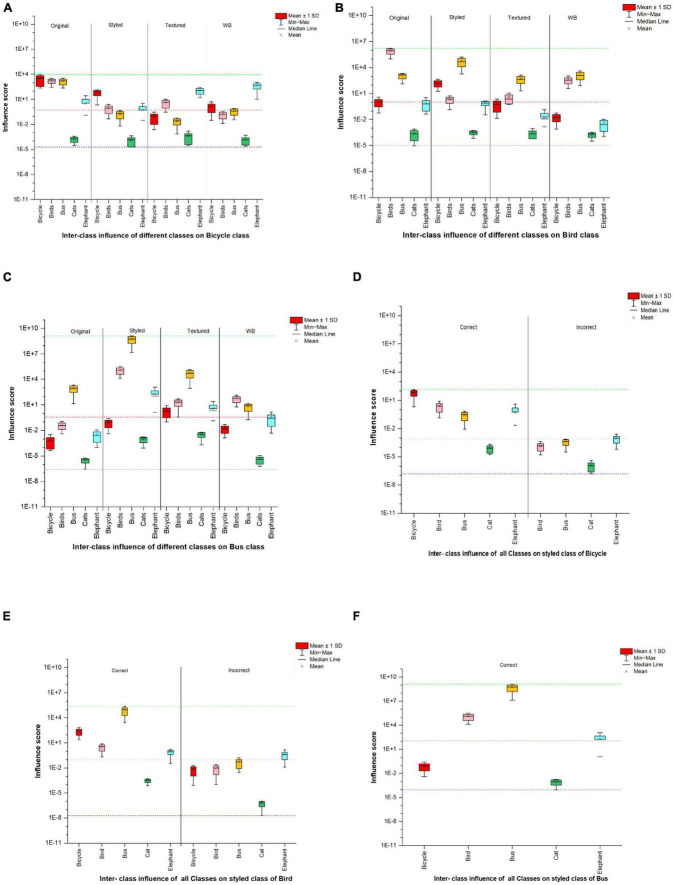
Results of inter-class influence score **(A)** influence of training set of different classes on testing set of bicycle class, **(B)** influence of training set of different classes on testing set of bird class, **(C)** influence of training set of different classes on testing set of bus class. Parts **(D,E)** shows the split in the corresponding styled image dataset showing correct and incorrect classifications, whereas in panel **(F)** all classes were correctly able to classify styled buses and hence no incorrect classification is given. Red dotted lines show the median, whereas the green and blue line show the min-max range of influence scores of the whole dataset. The images with influence score below the positive median range of the training set were incorrectly classified, whereas those above or are within the range of their intra-class influence are correctly classified.

Since the network was able to correctly classify most of the styled images (Table 1) we divided the styled image dataset to see it’s effect of the influence score on correct and incorrect class predictions. We further performed inter-class experiments to evaluate how the training set of one class influences the testing set of the other classes in terms of network’s class prediction capability. For demonstration purpose we show the results of inter-class evaluation for three classes and the corresponding dataset split for style images with correct and incorrect classification in Figure 7. The styling of images alters their appearance, making it difficult for the network to identify the original image features and use them for its decision. This network can provide some correct classification results in spite of the fluctuation in the layer-wise influence scores (Table 1), which is also reflected in the dataset split for styled images (Figures 4, 7). The model made a correct prediction when the influence score showed comparable fluctuation between the Intra-class scores of the original (control) images or between the range calculated for the original images of the inter-classes. However, incorrect predictions were made when the influence score were more dispersed from their corresponding control images.

#### 4.2.3. Analysis on images with white background

**FIGURE 8:**
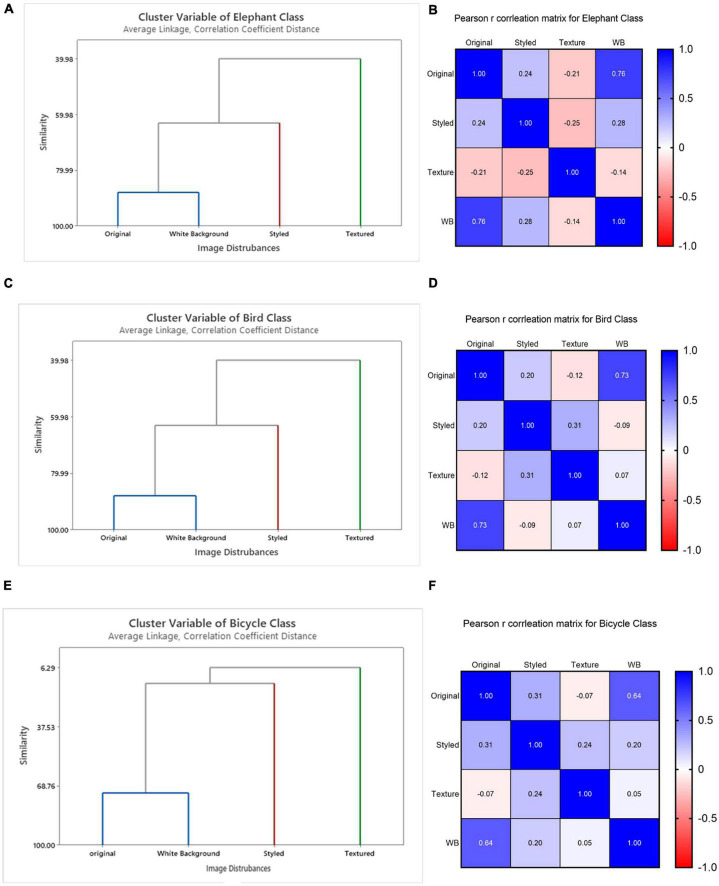
Layer-wise intra-class cluster analysis of the influence score on VGG16 network shown for three classes bicycle, bird, and elephant class. Parts **(A,C,E)** show three clusters, evaluated by Pearson’s distance correlation based on *I*_*avg*_. The original and white background images were determined as one cluster in terms of their visual similarity of features while style-textured images were identified as separate clusters based on dissimilarity from their controlled groups. Panels **(B,D,F)** shows their corresponding correlation matrix based on intra-class average layer-wise influence scores (*I*_*l*_*avg*_) for the above classes.

From our results of influential images and class prediction, we found that original and white background images have a close resemblance in terms of the influential images. To verify this resemblance, we performed hierarchical cluster analysis based on the layer-wise feature vectors calculated earlier in the section. As an example of this analysis, we show the results for three classes as well as their Pearson’s distance correlation matrices to interpret the results shown in Figure 8.

**FIGURE 9:**
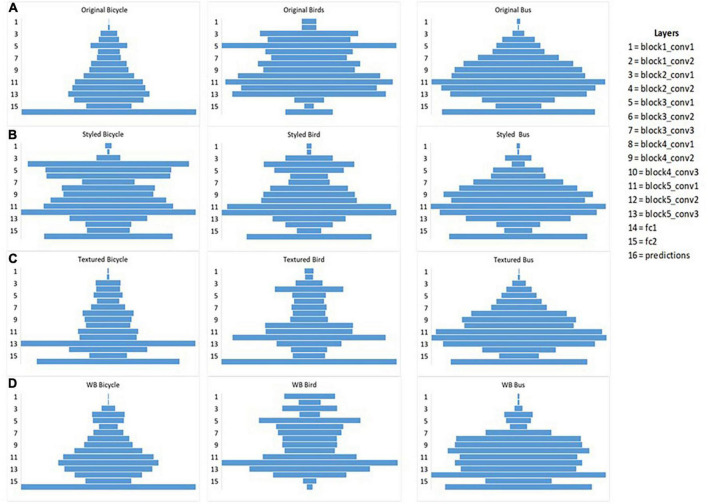
Layer-wise fluctuation of influence score shown for three classes bicycle, bus, and bird **(A)** layer-wise influence score for original images, **(B)** layer-wise influence score for styled images, **(C)** layer-wise influence score for textured images **(D)** layer-wise influence score for white background images. In the cases presented above, styled images fluctuate mostly in the middle layers, whereas for textured images the higher peaks are toward the last layers whereas for white background images the influence of object is more pronounced throughout similar to influence of its corresponding original images.

We identified three clusters: original and white background as one cluster shown in blue, styled in red and textured in green (Figure 8). The color of the cluster variables is assigned depending on how similar/dissimilar the observations are to better visualize the common characteristics in clustering. We assigned blue color to show grouping between original and white background images, red color for styled and green color for textured images. The similarity is determined based on the global influence score calculated for each intra-classes using *I_avg_*. To further learn about the common patterns that the model considers during decision making, we identified mean influence score of disturbed images at individual layers using *I*_*l_ avg*_. For demonstration purpose we present the results of three classes showing the mean influence score of different layers of all types of images as shown below in Figure 9. It was found that the layer-wise influence scores between the original and white-background images were highly similar, making the actual object in the image useful as a learned representation for prediction (Table 1). The layer-wise influence scores of the styled images fluctuated more toward the middle layers, indicating that the deeper layers observe abstract representations compared to their controlled groups. By contrast, the network identifies no visual similarity between the images with added texture and their corresponding original images. As a result, textured images show high peaks of layer-wise influence scores toward the last layers. Thus, embedded texture with high peaks at last layers tend to represent the learned texture as also shown in Table 3 where classification is made on the added texture. Thus, textured images formed a separate cluster and exhibited a negative influence, indicating a high degree of dissimilarity between layer-wise influence scores.

#### 4.2.4. Bi-directional interpretation of influence scores via Shapley values

**FIGURE 10:**
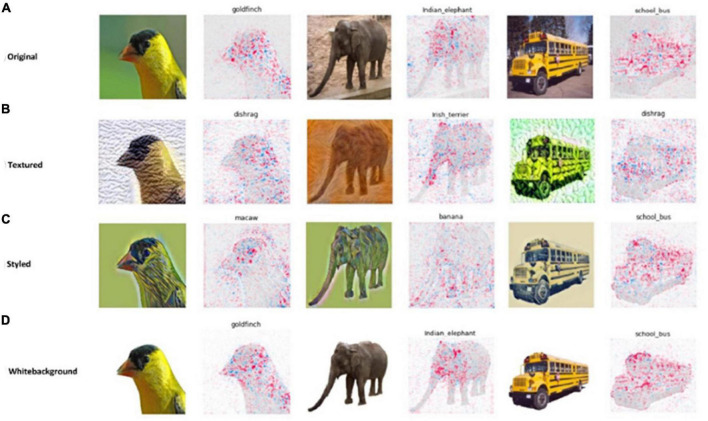
Shapley-value based influential regions of images with disturbances, **(A)** original **(B)** textured **(C)** styled, and **(D)** white background images.

To further evaluate and interpret the learned representations, we identified Shapley value-based (Lundberg and Lee, 2017) influential regions between different types of test inputs (Figure 10). Here, we did not calculate layer-wise Shapley values but only considered the test images to see which image regions were important for the network using our previous work (Aamir et al., 2022). The reason for this analysis is that we wanted to identify what the network looks at in making its decision. We considered a region to be influential if the Shapley values in that region were high (marked by red color in the Figure 10). As shown by the results of the clustering method, the original and white background images are grouped together, and the styled and textured images are grouped separately, this is also indicated in the Shapley values interpretation (Figure 10).

## 5. Discussion and conclusion

The interpretation of the reasons why a machine learning model arrived at a particular decision are still not understood, particularly for Deep Neural Networks, which are often referred to as “Black Box” models. Finding good reasons and making model decisions transparent are challenging tasks unless we know the underlying learned representations. We developed a layer-wise interpretability approach in order to increase transparency in network predictions based on intra-class layer-wise influence scores calculated from training images onto their corresponding test images with disturbances. According to Figures 10B, C, the influential regions of the styled and textured images are larger and more dispersed than those of the original images. Adding style or texture changes the visual appearance of the image and a pre-trained model, such as the one used in our study, is unable to make correct predictions. According to Hinton et al. (2015) and Kriegeskorte (2015), CNNs use a combination of low level as well as high-level complex features to make their decisions. Adding disturbances in images, as in our case, changes the visual appearance affecting the prediction capability of the model. Another reason for the misclassification of textured and styled images could be that CNN models rely on the shape of the object and tend to ignore color information associated with it (Ritter et al., 2017). In our study, we have also observed that removing the background and adding a white color background does not promote misclassification. However, distorted shape boundaries due to style and added texture made it impossible to identify low level features and the network failed to correctly classify the disturbed images. Specifically, the textured images were always classified based on the texture pattern added on the content image. The texture bias in the pre-trained CNN that we observed through the influence score has also been identified in the work of Geirhos et al. (2018) and Hermann et al. (2020) and retraining targeted layers may help to address this bias. Therefore, fine-tuning individual layers is more efficient than retraining the entire network. We demonstrated this using the VGG-16 network but this approach can be used for other pre-trained convolutional neural networks, because the overall functionality of all convolutional networks remains same. Since, our method is *post hoc* interpretable, analyzing networks with fewer layers is easier to interpret compared to very deep networks.

The work of Koh and Liang (2017) considers the entire parameter set of the model to identify influential images of the (training, testing) dataset and mostly works with adversarial images. However, giving reasons for the identified influential images and features that make the images influential over the others or interpretation of neural network decisions based on the influential images was not the scope of their paper. We therefore built on the idea of identifying influential images, and firstly modified our images with disturbances and we do not consider adversarial images in our dataset. The reason for adding disturbances in images is to make model decisions more transparent, hence going a step further to explore individual layers. We have explored the layer-wise influences of individual (training, testing) ImageNet images as well as the influences of modified images. This way we were able to identify which layers can be retrained or fine-tuned to deal with images that are slightly modified but belong to the same intra classes. This approach could in particular be useful in medical domain to identify normal and slightly distorted or modified images this way potentially helping in early diagnostics of various diseases. We calculated the compound influence (I_{total}) score as well as layer-wise scores to identify transparent solutions of influential (training, testing) images. We observed that our compound and layer-wise influence scores were somewhat related but the compound influence of the layers did not give decisive results on the modified images as compared to the layer-wise analysis. Hence, there is definitely a clear gain in obtaining results of local and more precise influential images compared to global and more generalized network descriptors, for which we have provided evidence by the different analyses shown in the paper. The approach presented here can be seen as a step toward providing deeper insights and transparency regarding the internal states of deep learning models. Several types of disturbed input images were tested in order to observe the effects of our method on class predictions. We have found that a positive layer-wise influence score range of the training instance provides information about why there are correct or incorrect network decisions. In addition, identifying specific layers where the disturbed images’ influence scores are most noticeable can be fined tuned to improve the correct predictions. Considering styled images, we observed correct class prediction when the influence score ranges in between the intra-class scores of the original images or between the ranges calculated for the original images of the inter-classes. The removal of background, however, does not have much effect on the correct class prediction and the foreground object alone is sufficient for the correct classification. This is in spite of the fact that the network strongly relies on textural cues for its classification and a sudden drop in classification accuracy is observed as soon as the percentage of texture is increased. Thus, this suggests that one can overcome the texture bias and retrain the target layers to improve the predictability of the deep network models.

Currently in our study, we have proposed a method that can make the decisions of a network transparent by providing layer-wise influence scores. We tested our methodology by adding disturbances to images taken from the ImageNet dataset. It might be useful to further improve our method and make it more robust by testing it on other publicly available datasets. This would help to provide a more refined contribution of identifying the role of disturbances in individual layers of the networks. This information could then be used to fine-tune and/or retrain the network in order to avoid biased decisions. Furthermore, our method is constrained by using only a limited number of image disturbances. In future work this limitation can be overcome by including other more complex disturbance patterns or by using test images without any texture, for example, just simple shape outlines of the objects. Other future work may involve using this layer-wise influence score to obtain more transparent solutions to image classification problems in the medical domain. In particular, we believe that we could improve classification accuracy and interpret and debug the model to achieve better results, which should be a promising direction for future research.

## Data availability statement

The original contributions presented in this study are included in the article/Supplementary material, further inquiries can be directed to the corresponding author.

## Author contributions

AA contributed the main idea, designed the algorithm, performed all the data analysis, and wrote the first draft of the manuscript. MT and FW supervised, reviewed, and edited the manuscript. AA, MT, and FW edited the final manuscript. All authors contributed to the article and approved the submitted version.
