# Supplementary figures and images for: Interpreting the decisions of CNNs via influence functions
Source: Front Comput Neurosci. 2023 Jul 26;17:1172883. doi: 10.3389/fncom.2023.1172883 (PMC10410673; doi:10.3389/fncom.2023.1172883)

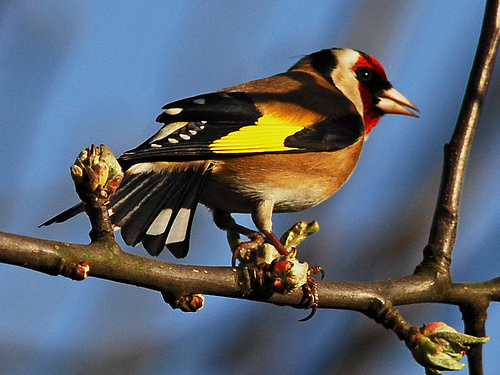

Supplement: Supplementary file 1 [file Data_Sheet_1.ZIP › Sample-dataset/o/1.JPEG]

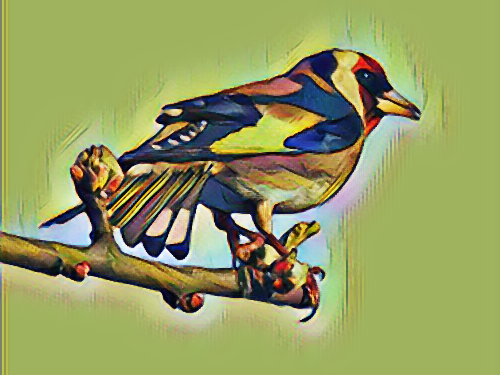

Supplement: Supplementary file 1 [file Data_Sheet_1.ZIP › Sample-dataset/o/1.png]

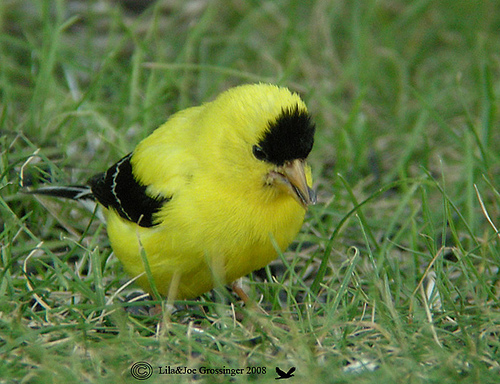

Supplement: Supplementary file 1 [file Data_Sheet_1.ZIP › Sample-dataset/o/2.JPEG]

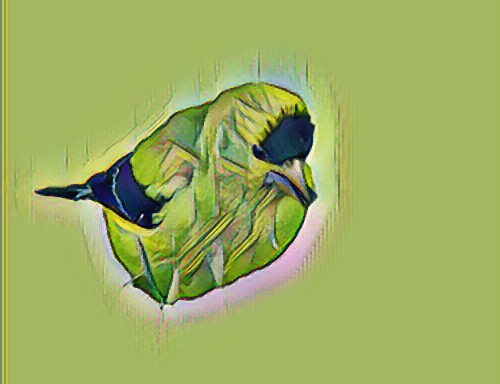

Supplement: Supplementary file 1 [file Data_Sheet_1.ZIP › Sample-dataset/o/2.png]

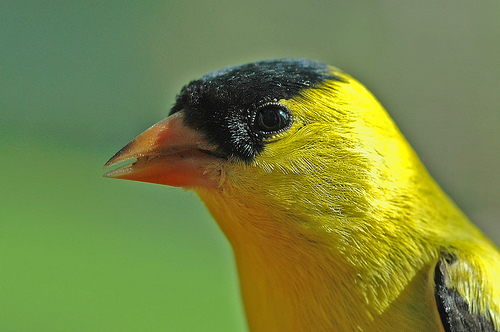

Supplement: Supplementary file 1 [file Data_Sheet_1.ZIP › Sample-dataset/o/3.JPEG]

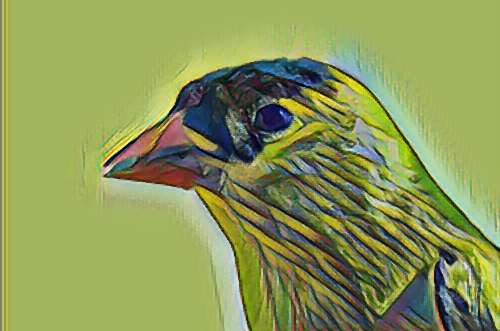

Supplement: Supplementary file 1 [file Data_Sheet_1.ZIP › Sample-dataset/o/3.png]

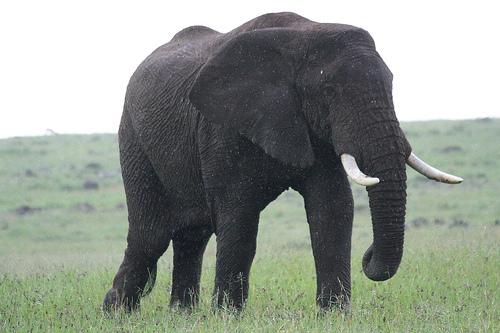

Supplement: Supplementary file 1 [file Data_Sheet_1.ZIP › Sample-dataset/o/4.JPEG]

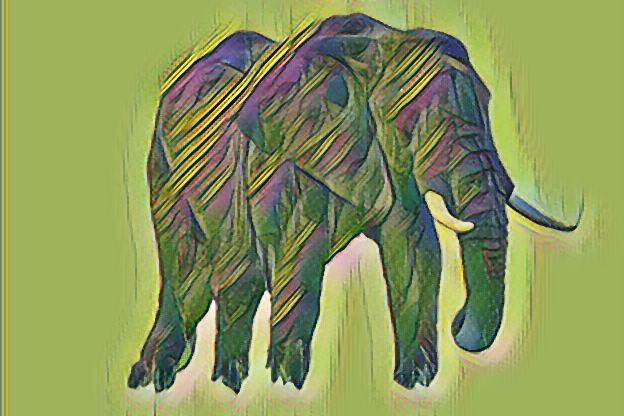

Supplement: Supplementary file 1 [file Data_Sheet_1.ZIP › Sample-dataset/o/4.png]

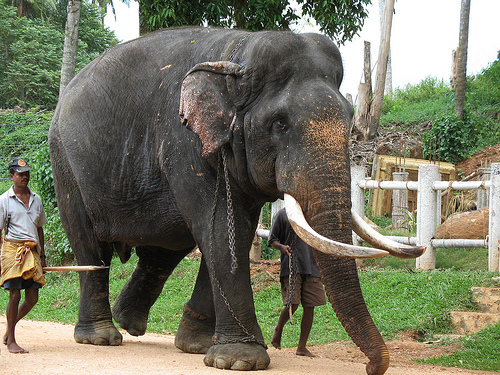

Supplement: Supplementary file 1 [file Data_Sheet_1.ZIP › Sample-dataset/o/5.JPEG]

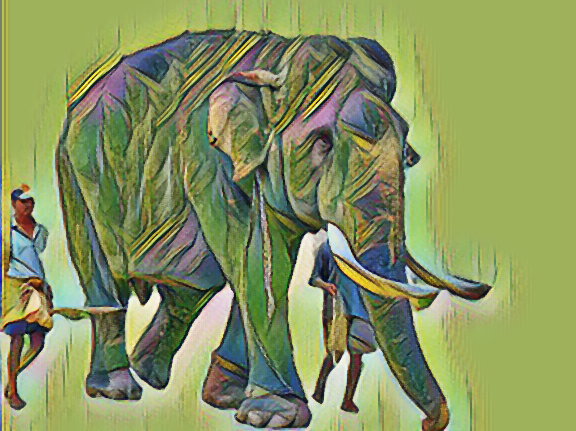

Supplement: Supplementary file 1 [file Data_Sheet_1.ZIP › Sample-dataset/o/5.png]

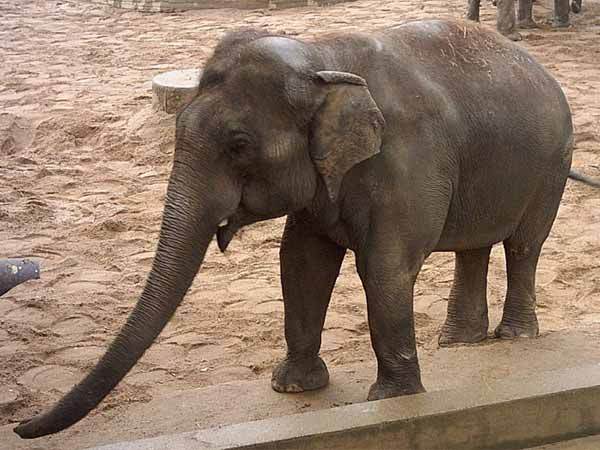

Supplement: Supplementary file 1 [file Data_Sheet_1.ZIP › Sample-dataset/o/6.JPEG]

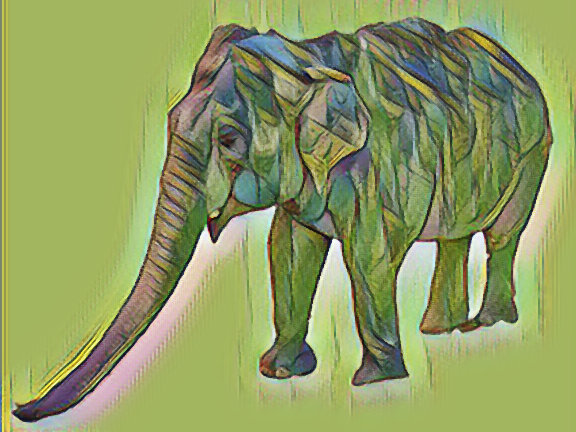

Supplement: Supplementary file 1 [file Data_Sheet_1.ZIP › Sample-dataset/o/6.png]

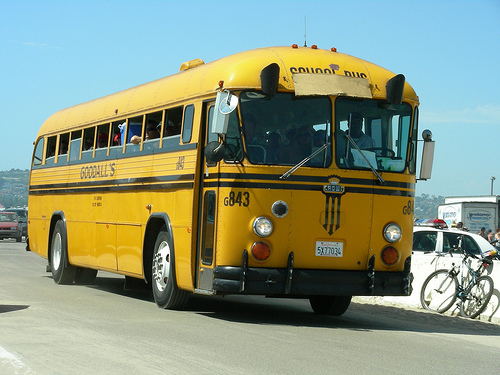

Supplement: Supplementary file 1 [file Data_Sheet_1.ZIP › Sample-dataset/o/7.JPEG]

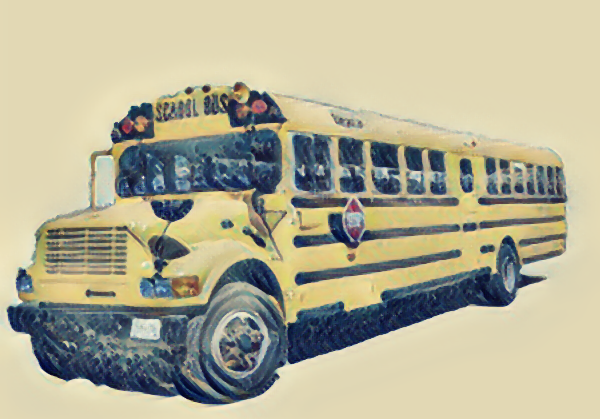

Supplement: Supplementary file 1 [file Data_Sheet_1.ZIP › Sample-dataset/o/7.png]

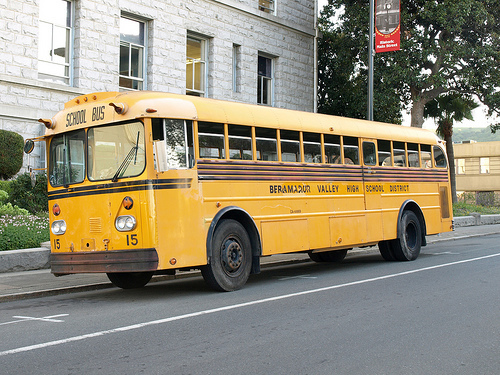

Supplement: Supplementary file 1 [file Data_Sheet_1.ZIP › Sample-dataset/o/8.JPEG]

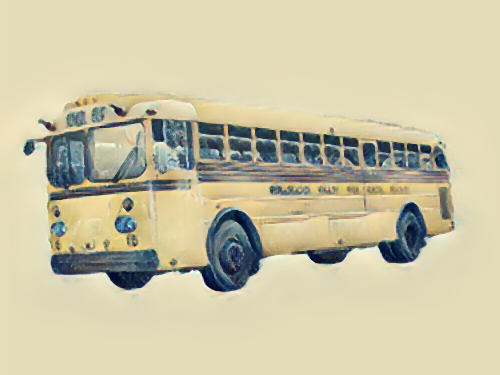

Supplement: Supplementary file 1 [file Data_Sheet_1.ZIP › Sample-dataset/o/8.png]

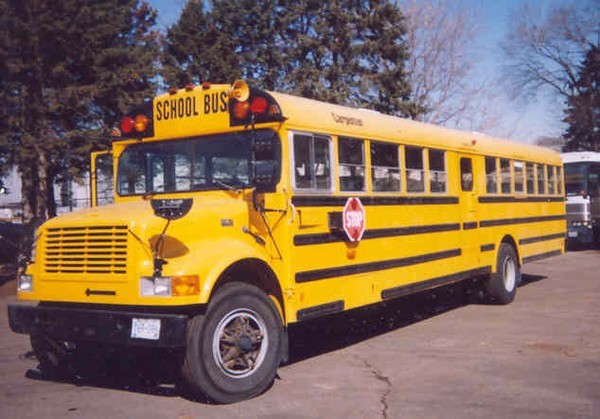

Supplement: Supplementary file 1 [file Data_Sheet_1.ZIP › Sample-dataset/o/9.JPEG]

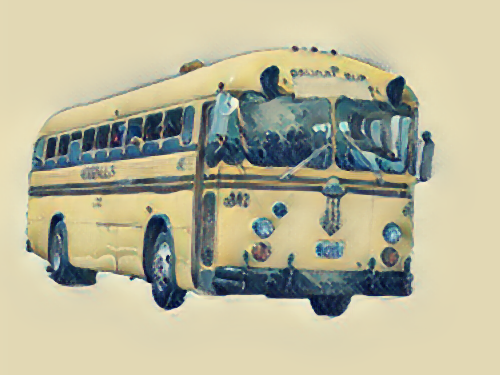

Supplement: Supplementary file 1 [file Data_Sheet_1.ZIP › Sample-dataset/o/9.png]

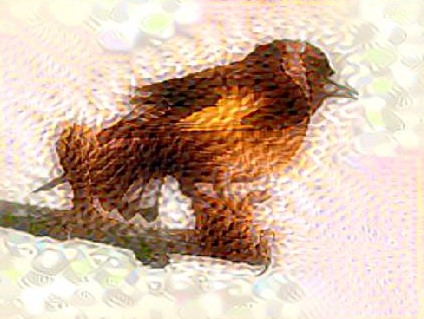

Supplement: Supplementary file 1 [file Data_Sheet_1.ZIP › Sample-dataset/t/1.jpg]

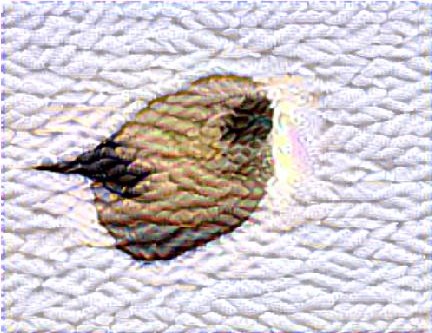

Supplement: Supplementary file 1 [file Data_Sheet_1.ZIP › Sample-dataset/t/2.jpg]

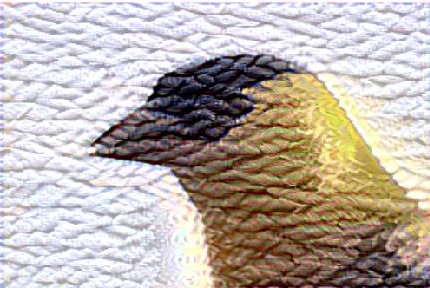

Supplement: Supplementary file 1 [file Data_Sheet_1.ZIP › Sample-dataset/t/3.jpg]

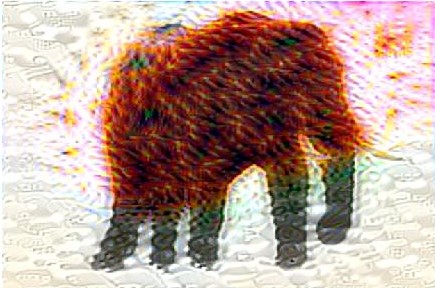

Supplement: Supplementary file 1 [file Data_Sheet_1.ZIP › Sample-dataset/t/4.jpg]

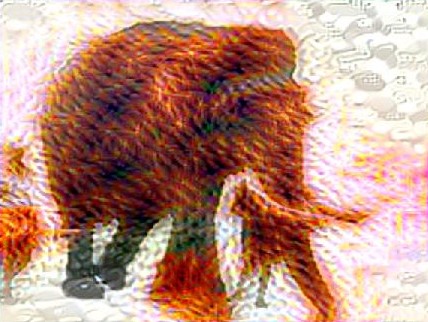

Supplement: Supplementary file 1 [file Data_Sheet_1.ZIP › Sample-dataset/t/5.jpg]

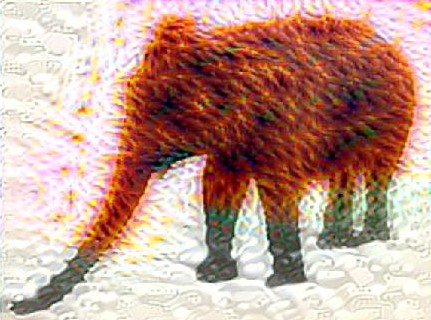

Supplement: Supplementary file 1 [file Data_Sheet_1.ZIP › Sample-dataset/t/6.jpg]

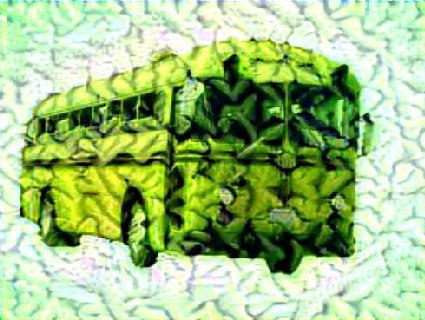

Supplement: Supplementary file 1 [file Data_Sheet_1.ZIP › Sample-dataset/t/7.jpg]

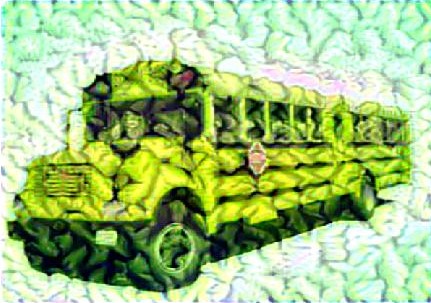

Supplement: Supplementary file 1 [file Data_Sheet_1.ZIP › Sample-dataset/t/8.jpg]

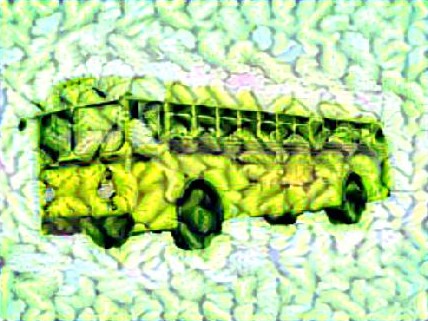

Supplement: Supplementary file 1 [file Data_Sheet_1.ZIP › Sample-dataset/t/9.jpg]

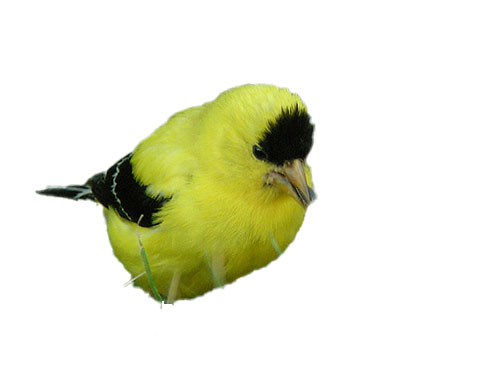

Supplement: Supplementary file 1 [file Data_Sheet_1.ZIP › Sample-dataset/w/1.jpg]

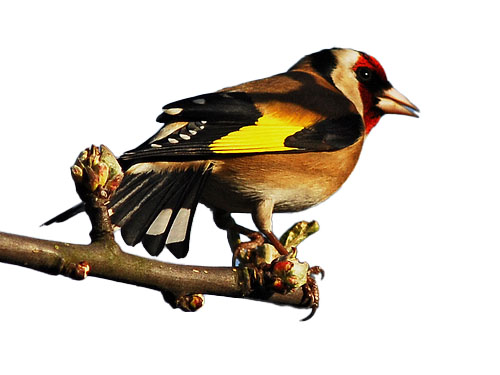

Supplement: Supplementary file 1 [file Data_Sheet_1.ZIP › Sample-dataset/w/2.jpg]

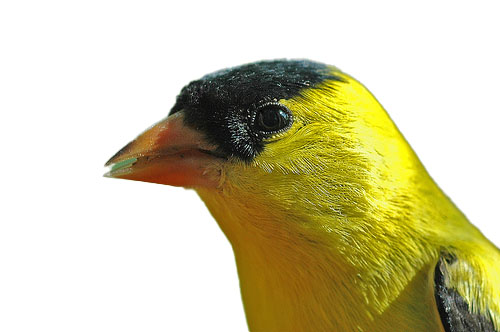

Supplement: Supplementary file 1 [file Data_Sheet_1.ZIP › Sample-dataset/w/3.jpg]

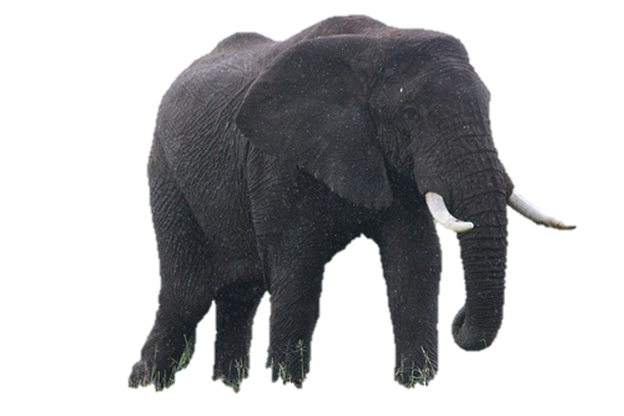

Supplement: Supplementary file 1 [file Data_Sheet_1.ZIP › Sample-dataset/w/4.png]

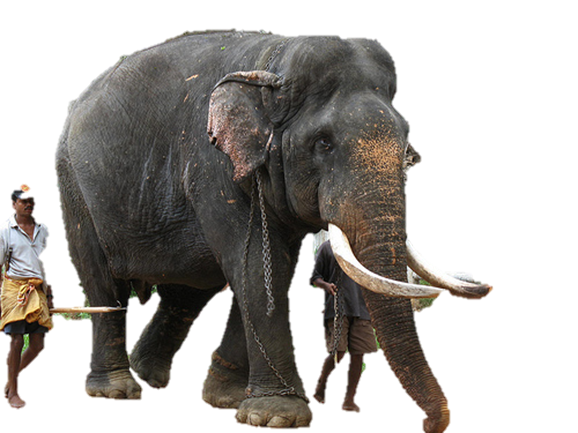

Supplement: Supplementary file 1 [file Data_Sheet_1.ZIP › Sample-dataset/w/5.png]

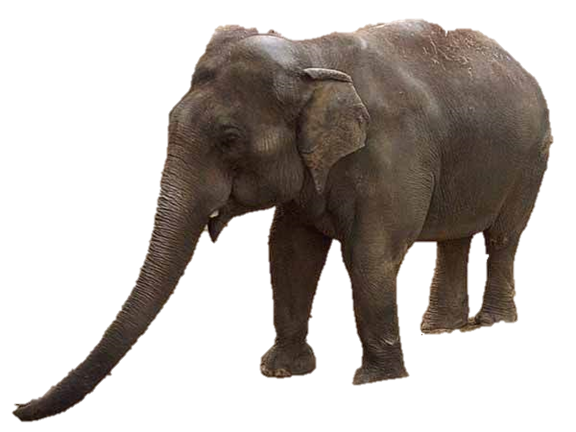

Supplement: Supplementary file 1 [file Data_Sheet_1.ZIP › Sample-dataset/w/6.png]

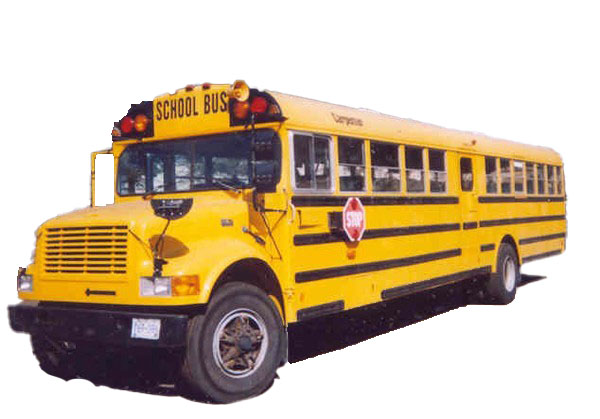

Supplement: Supplementary file 1 [file Data_Sheet_1.ZIP › Sample-dataset/w/7.jpg]

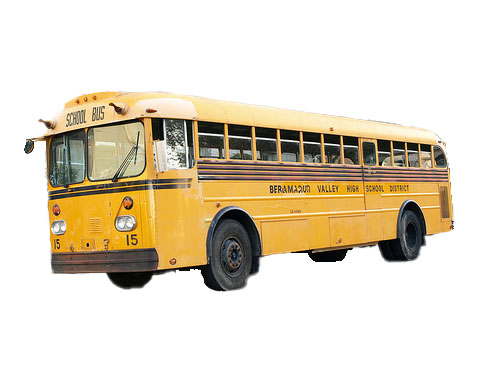

Supplement: Supplementary file 1 [file Data_Sheet_1.ZIP › Sample-dataset/w/8.jpg]

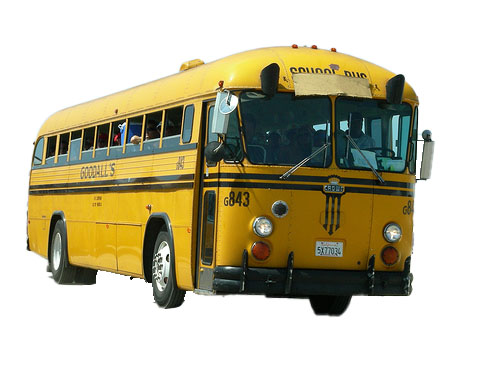

Supplement: Supplementary file 1 [file Data_Sheet_1.ZIP › Sample-dataset/w/9.jpg]
